# Supplementary material for: Measuring effects of ivermectin-treated cattle on potential malaria vectors in Vietnam: A cluster-randomized trial
Source: PLoS Negl Trop Dis. 2024 Apr 29;18(4):e0012014. doi: 10.1371/journal.pntd.0012014 (PMC11098492; doi:10.1371/journal.pntd.0012014)
Supplement: S1 Text — Fig A in S1 Text. Location of study villages, ZAIVE trial, Sept 2019, Gai Lai Vietnam. (note: C indicates control village; T indicates ivermectin treatment (intervention) village). Table A in S1 Text. Reported malaria cases per study village in 2018, ZAIVE trial, Central Vietnam. Table B in S1. Spatial variation in total anophelines captured via cattle-baited traps (pre-intervention, pooled by study village, N = 6), Sept 2019, Gai Lai Vietnam. Table C in S1. Temporal variation in total anopheline captured via cattle-baited traps pre-intervention, pooled by trap-night, N = 6), Sept 2019, Central Vietnam. Fig B in S1 Text. Coefficient of variation in nightly total anophelines captured via cattle-baited traps (pre-intervention, pooled by trap-night, N = 6), Sept 2019, Central Vietnam. Fig C in S1 Text. Comparison of distribution of trapping-night counts, ZAIVE trial, Sept 2019, Central Vietnam. Fig D in S1 Text. GEE model residuals, negative binomial distribution, stratified by treatment arm, ZAIVE trial, Sept 2019, Central Vietnam. Fig E in S1 Text. Difference-in-differences analysis, using general estimating equations (GEE) model with negative binomial distribution, stratified by treatment arm, ZAIVE trial, Sept 2019, Central Vietnam. Table D in S1 Text. Alternative model specification for primary outcome, for ZAIVE trial, Sept 2019, Gai Lai Vietnam. Table E in S1 Text. Study power calculations for pre- and post-interventional designs with entomological outcomes, based on ZAIVE field data. (CV = coefficient of variation). ZAIVE trial, Sept 2019, Gai Lai Vietnam). Table F in S1 Text. Simulation-based study design power calculations, parametrized from IMPE field surveillance data (as in main text), ZAIVE trial, Sept 2019, Gai Lai Vietnam. Fig F in S1 Text. Total trapping numbers, most common Anopheles species per trap-night night before and after intervention; Sept 2019, Gai Lai Vietnam. Fig G in S1 Text. Brillouin’s diversity index calculated for each day of mosquito [file pntd.0012014.s001.pdf]

ZAIVE trial, S1 Text (Supplemental Information).

I. Map of study areas 1
II. Village demographics, malaria cases, and cattle census 2
III. Quantifying spatio-temporal variation in anopheline counts from cattle-baited traps 2
IV. Model residuals and alternative model specifications 4
V. Estimating power for future related studies 7
VI. Expanded entomological measures 8
References 11

I. Map of study areas

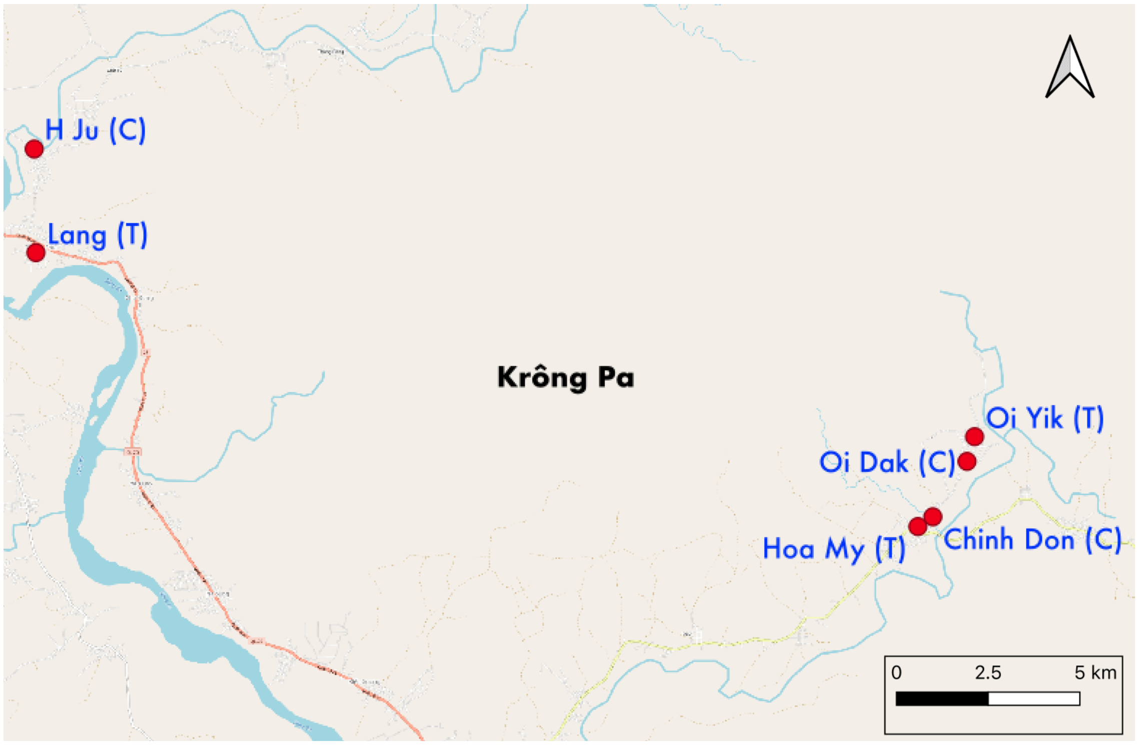

Figure A: Location of study villages, ZAIVE trial, Sept 2019, Central Vietnam. (note: C indicates control village; T indicates ivermectin treatment (intervention) village).

Note: Map data: Openstreet map, https://osmfoundation.org/wiki/Licence (Accessed Nov 1, 2023).

II. Village demographics, malaria cases, and cattle census

Village	Total population, N	Village health workers, N	*Plasmodium falciparum* cases, N	*P. vivax* cases, N	API, all species (per 1000 population)
**HM (Hòa Mỹ)**	508	3	0	0	0
**OJ (Ơi Jit)**	542	3	4	2	11.1
**CD (Chính Don)**	325	2	10	6	49.2
**OD (Ơi Đăk)**	334	2	2	0	6.0
**HY (H Yú)**	618	2	1	4	8.1
**HL (H Lang)**	1891	3	6	8	7.4

Table A: Reported malaria cases per village in 2018, ZAIVE trial, Central Vietnam. (API= annual parasitemia index).

III. Quantifying spatio-temporal variation in anopheline counts from cattle-baited traps

Larger-than-expected variability in trapping counts were observed both within- and between-villages in the ZAIVE trial. To quantify this spatial and temporal variability to inform future studies, the mean captures and coefficient of variation (with BCa bootstrapped 95% CIs; Stata 17; 2,000 replications) for the non-intervention period (trapping-nights 1 to 6) are presented below.

Village (trapping nights 1-6 combined)	mean	95% CI	CV	95% CI
1. H Yú	43.83	36.17 – 50.33	0.211	0.077 – 0.270
2. H Lang	51.83	35.0 – 68.67	0.470	0.277 – 0.642
3. Chính Đơn	153.83	113.17 – 214.33	0.438	0.301 – 0.643
4. Hòa Mỹ	60.67	36.67 – 88.33	0.597	0.309 – 0.926
5. Ơi Đăk	114.50	74.33 – 171.83	0.551	0.349 – 0.843
6. Ơi Jit	153.17	117.5 – 186.5	0.302	0.196 – 0.492
**Overall**	96.31	78.44 – 119.33	0.658	0.556 - 0.812

Table B. Spatial variation in total anophelines captured via cattle-baited traps (pre-intervention, pooled by *study village*, N= 6), Sept 2019, Gai Lai Vietnam.

Trapping night (Villages 1-6 combined)	mean	95% CI	CV	95% CI
1. 10 Sept 2019	79.17	40.67 – 129.83	0.743	0.511 – 1.05
2. 12 Sept 2019	73.33	59.0 – 97.67	0.357	0.193 – 0.509
3. 14 Sept 2019	138.5	82.67 – 209.83	0.634	0.402 – 1.19
4. 16 Sept 2019	117.5	60.83 – 179.17	0.724	0.373 – 1.11
5. 18 Sept 2019	98.33	50.17 – 139.67	0.622	0.359 – 1.04
6. 20 Sept 2019	71.0	45.17 – 88.33	0.410	0.184 – 0.862

Table C. Temporal variation in total anopheline captured via cattle-baited traps pre-intervention, pooled by *trap-night*, N=6), Sept 2019, Central Vietnam.

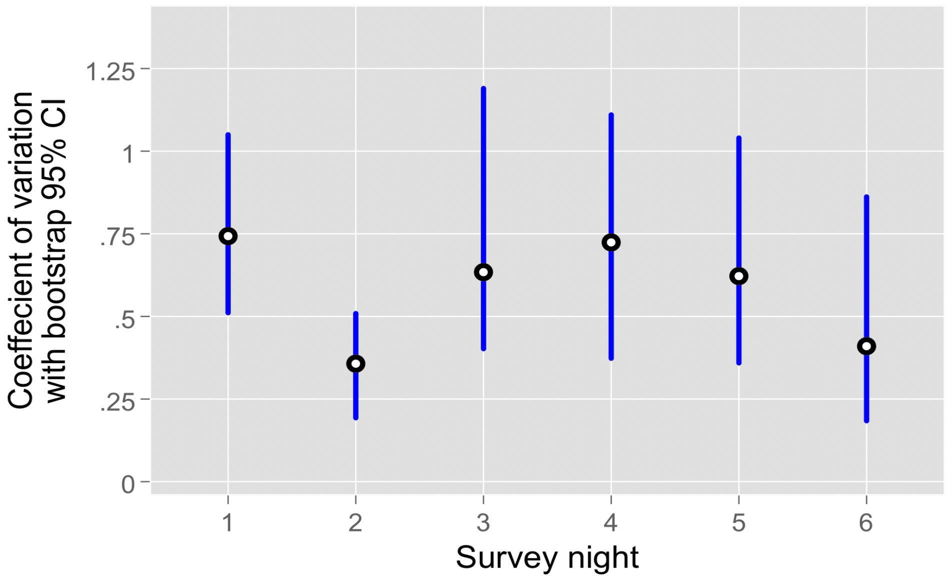

Figure B. Coefficient of variation in nightly total anophelines captured via cattle-baited traps (pre-intervention, pooled by trap-night, N= 6), Sept 2019, Central Vietnam.

IV. Model residuals and alternative model specifications

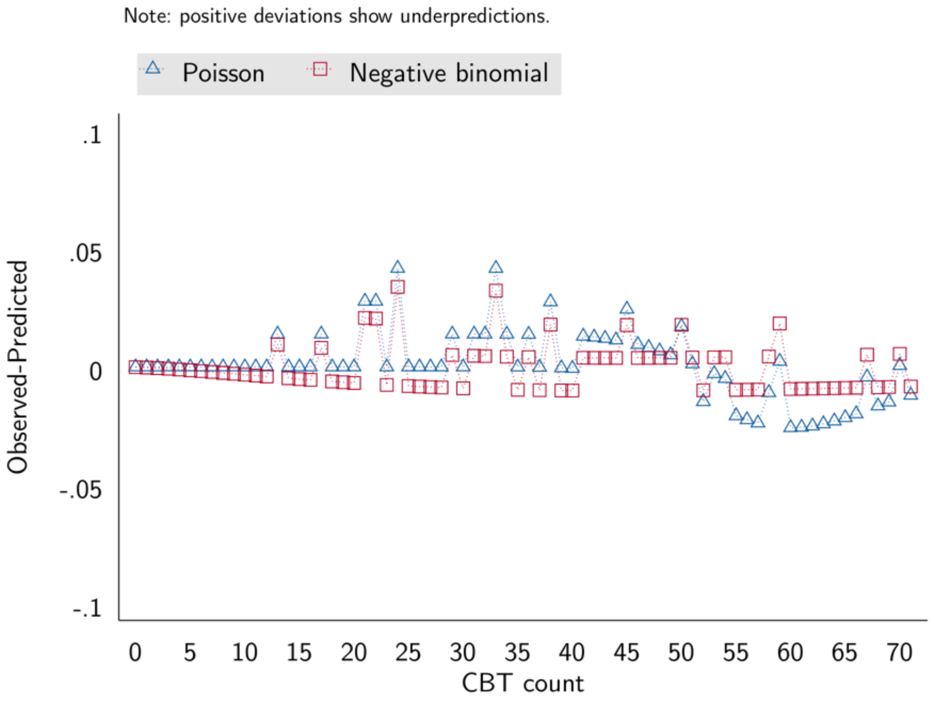

Figure C. Comparison of distribution of trapping-night counts, ZAIVE trial, Sept 2019, Central Vietnam.

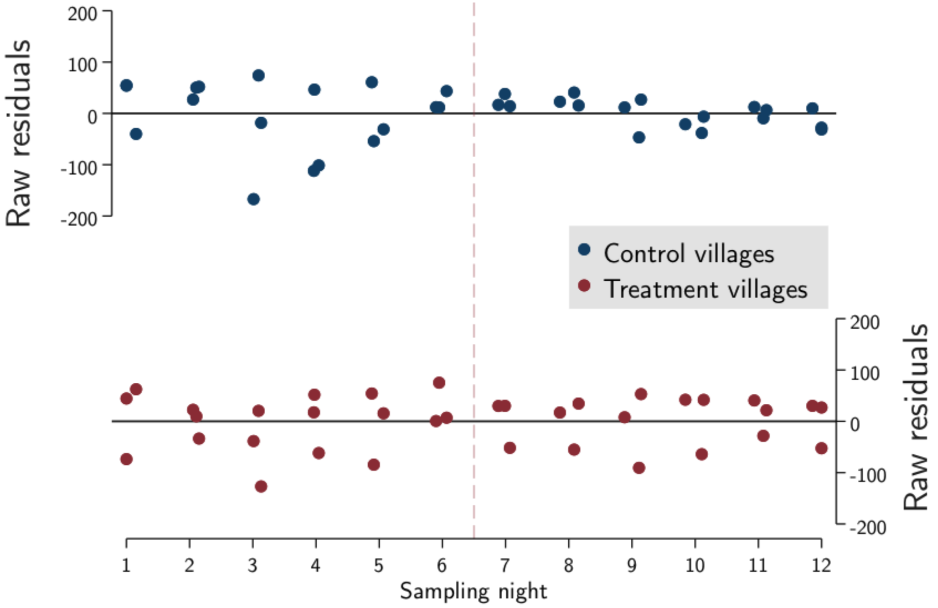

Figure D. GEE model residuals, negative binomial distribution, stratified by treatment arm, ZAIVE trial, Sept 2019, Central Vietnam.

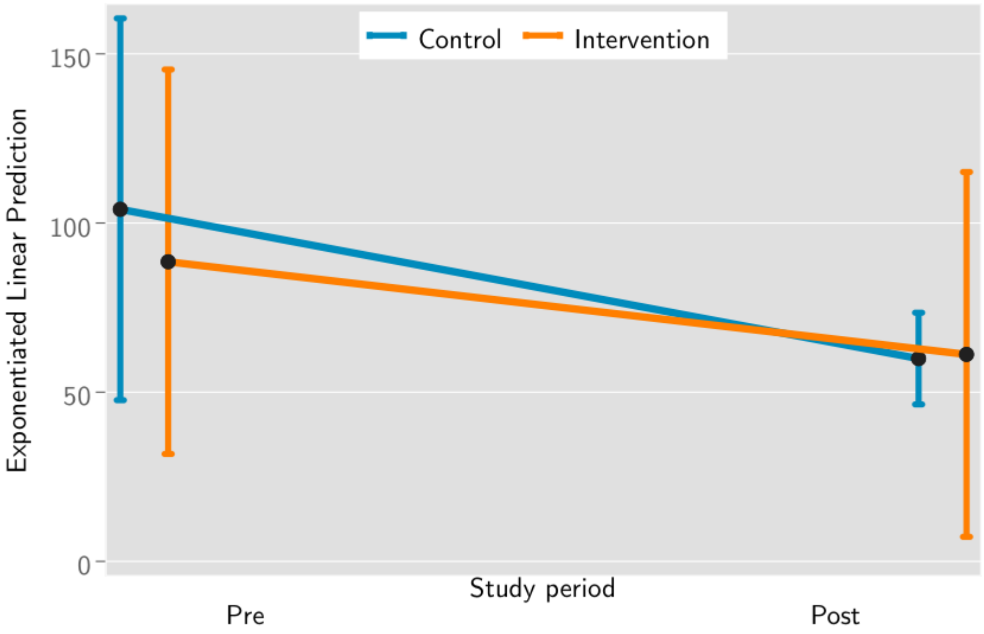

Figure E. Difference-in-difference analysis, using general estimating equations (GEE) model with negative binomial distribution, stratified by treatment arm, ZAIVE trial, Sept 2019, Central Vietnam.

	Model			
	(Protocol analysis; GEE, neg binomial; robust errors)	(GEE, Poisson; robust errors)	(GEE, neg binomial; bootstrap errors)	(Quantile regression for median; robust errors)
	IRR	IRR	IRR	Beta
**Intervention: Control arm**				
Coefficient	0.85	0.85	0.85	-15.00
95% CI	0.37, 1.97	0.37, 1.97	0.36, 2.03	-75.40, 45.40
p-value	0.71	0.71	0.72	0.62
**Post : pre period**				
Coefficient	0.58	0.58	0.58	-31.00
95% CI	0.31, 1.07	0.31, 1.07	0.30, 1.12	-82.79, 20.79
p-value	0.08	0.08	0.11	0.24
**Interaction term**				
Coefficient	1.20	1.20	1.20	1.00
95% CI	0.60, 2.39	0.60, 2.39	0.54, 2.67	-77.70, 79.70
p-value	0.61	0.61	0.66	0.98
**Intercept**				
Coefficient	104.06	104.06	104.06	90.00
95% CI	60.51, 178.95	60.51, 178.95	57.23, 189.19	43.68, 136.32
p-value	< 0.001	< 0.001	< 0.001	< 0.001

Table D. Alternative model specification for primary outcome, for ZAIVE trial, Sept 2019, Gai Lai Vietnam.

Quantile (median) regression for analysis of overdispersed outcomes. [1]

V. Estimating power for future related studies

Using the means and coefficients of variation from the six baseline surveys, simulation-based power calculations (Stata 17; 500 replicates) were performed to assess potential study power for future studies of similar design. All scenarios power to detect a 50% reduction in all anophelines, using a differences-in-differences design, and a GEE model with negative binomial distribution.

Data Scenario	Trapping nights, pre/post	Trapping stations per village	Mean captures per trap-night (table S4 above)	CV Estimate	Study power
**Pooled, all sites**	6/6	1	96	0.66	0.70
**Highest CV trap-night**	6/6	1	79	0.74	0.64
**Lowest CV trap-night**	6/6	1	73	0.36	0.98
**Highest mean trap-night**	6/6	1	139	0.63	0.71

Table E. Study power calculations for pre- and post-interventional designs with entomological outcomes, based on ZAIVE field data. (CV= coefficient of variation). ZAIVE trial, Sept 2019, Gai Lai Vietnam).

Trapping nights, pre/post	Trapping stations per village	Mean captures per trap-night	SD of trapping rates	Study power
**6/6**	1	35	20	0.79
**6/6**	**1**	**40**	**20**	**0.84**
**7/7**	1	35	20	0.84
**7/7**	1	40	20	0.90

Table F. Simulation-based study design power calculations, parametrized from IMPE field surveillance data (as in main text), ZAIVE trial, Sept 2019, Gai Lai Vietnam.

VI. Expanded entomological measures

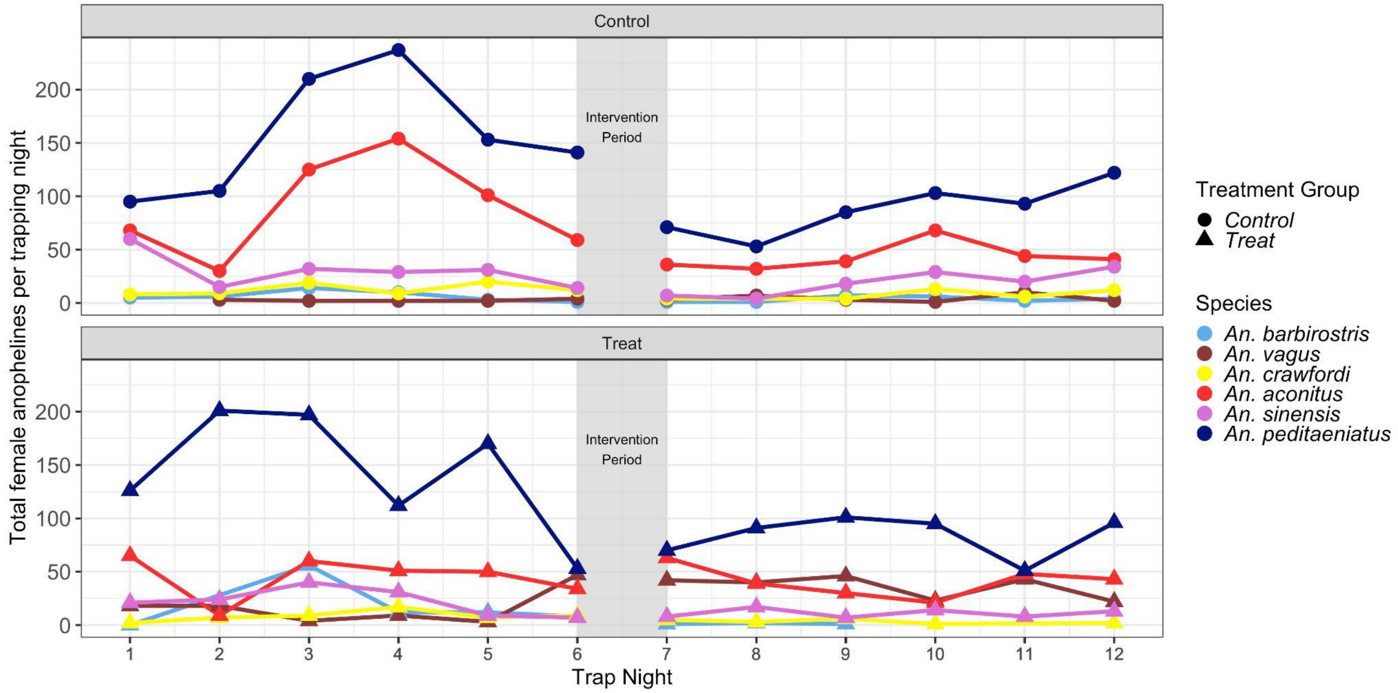

Figure F. Total trapping numbers, most common *Anopheles* species per trap-night night before and after intervention; Sept 2019, Gai Lai Vietnam.

Each main species is represented by one color. Dotted lines and triangles represent mosquitoes from villages that received IVM treatment for their cattle, and solid lines with circles represent mosquitoes captured in villages that were not treated with IVM.

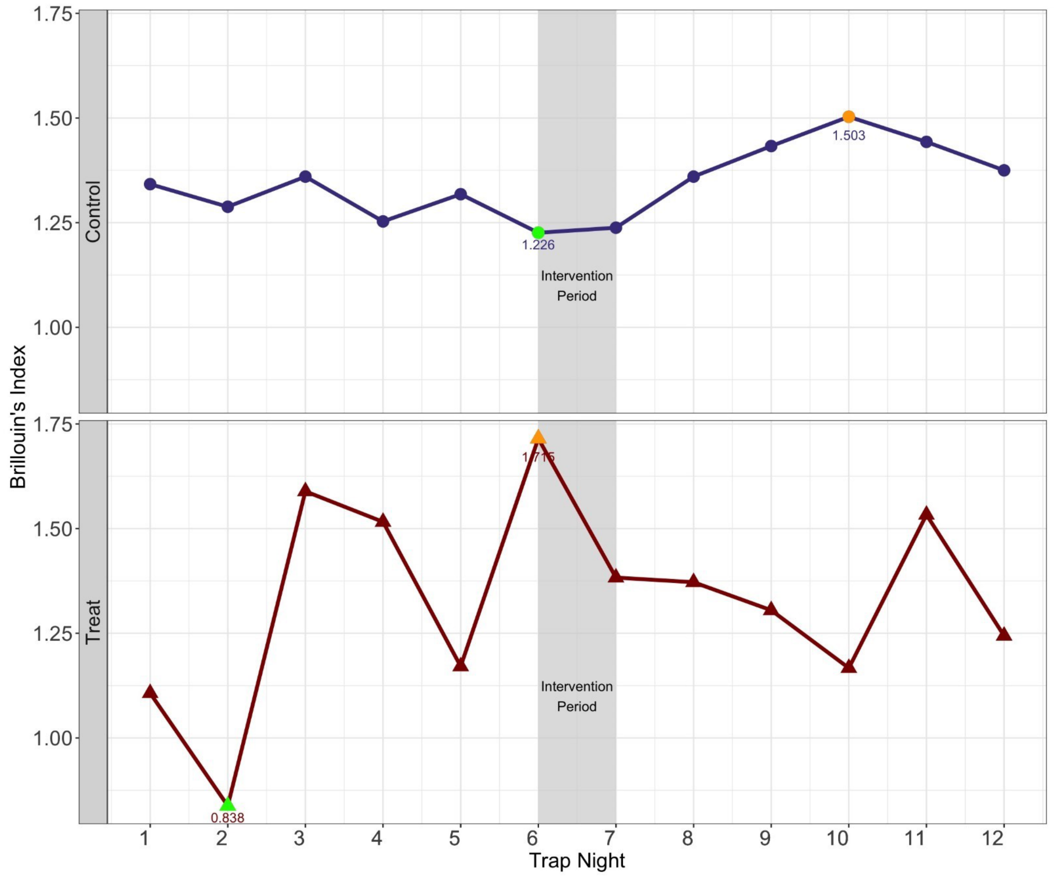

Figure G: Brillouin's diversity index calculated for each day of mosquito captures, ZAIVE trial, Sept 2019, Gai Lai Vietnam.

Brillouin's index was calculated by trap-night in each arm. Orange markers represent the maximum value and green markers represent the minimum value. Higher values indicate increased levels of diversity.

Species	Control mean difference	Treatment mean difference	t-test statistic	p-value for difference
Anopheles barbirostris	6.00	28.00	-0.84	0.48
An. vagus	-4.33	-39.00	0.93	0.45
An. crawfordi	11.33	11.33	0.00	1.00
An. aconitus	92.33	12.50	0.65	0.56
An. sinensis	23.00	21.67	0.04	0.97
An. peditaeniatus	138.00	118.33	0.23	0.83

Table G. Differences in pre- and-post-intervention trapping totals, by main anopheline species, ZAIVE trial, Sept 2019, Gai Lai Vietnam.

Time period	Treatment Arm	Village	Cattle Baited Trap, N	CDC light trap, N	Double-net traps, N	Human landing catch, N
post	Control	Chính Đơn (CD)	291	10	0	0
pre	Control	Chính Đơn (CD)	923	19	0	0
post	Treat	H Lang (HL)	245	7	0	0
pre	Treat	H Lang (HL)	311	1	0	0
post	Control	H Yú (HY)	340	4	0	0
pre	Control	H Yú (HY)	263	2	0	0
post	Treat	Hòa Mỹ (HM)	128	7	0	0
pre	Treat	Hòa Mỹ (HM)	364	11	0	0
post	Control	Ơi Đăk (OD)	448	9	0	0
pre	Control	Ơi Đăk (OD)	687	9	0	0
post	Treat	Ơi Jit (OJ)	730	17	0	0
pre	Treat	Ơi Jit (OJ)	919	17	0	0

Table H. Trap-night anopheline totals by trapping method, ZAIVE trial, Sept 2019, Gai Lai Vietnam.

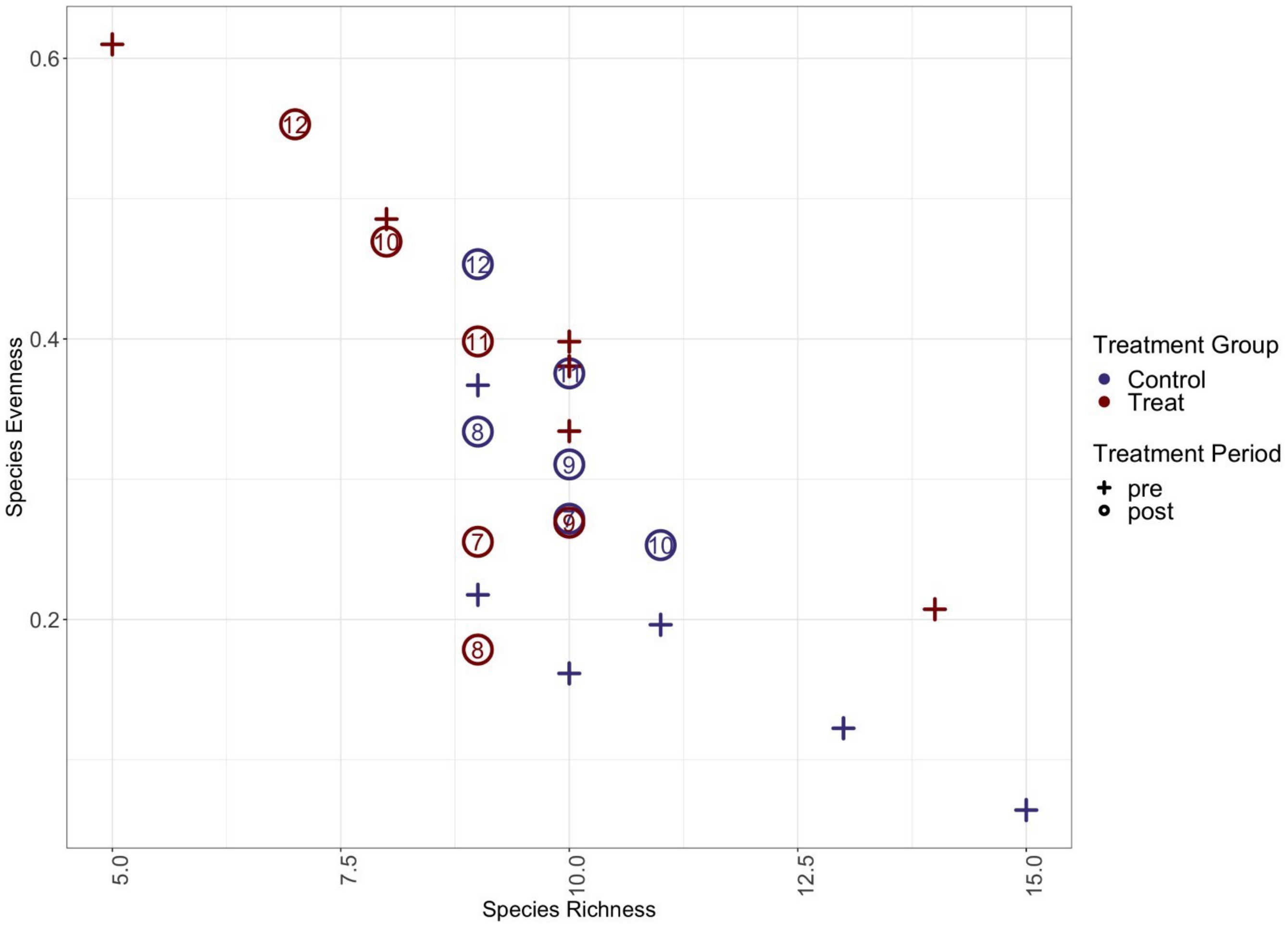

Figure H. Decomposition of diversity into richness and evenness components, by study arm, ZAIVE trial, Sept 2019, Gai Lai Vietnam.

This figure represents the calculated richness and evenness for each village on each date. Crosses represent the dates before treatment, and circles represent the dates after treatment. Numbers in circles represent the number of nights after treatment. Higher values on the y-axis indicate increased species diversity, and higher values on the x-axis represent increased species richness.

References

1. McGreevy KM, Lipsitz SR, Linder JA, Rimm E, Hoel DG. Using median regression to obtain adjusted estimates of central tendency for skewed laboratory and epidemiologic data. Clinical Chemistry. 2009;55: 165–169. doi:10.1373/clinchem.2008.106260
