# Supplementary material for: Measuring effects of ivermectin-treated cattle on potential malaria vectors in Vietnam: A cluster-randomized trial
Source: PLoS Negl Trop Dis. 2024 Apr 29;18(4):e0012014. doi: 10.1371/journal.pntd.0012014 (PMC11098492; doi:10.1371/journal.pntd.0012014)
Supplement: Attachment — Submitted filename: Response to Reviewers_PNTD-D-23-00193.pdf [file pntd.0012014.s003.pdf]

Response to reviewers, (PNTD-D-23-00193).

We thank the reviewers for their thoughtful and detailed review of this manuscript. We have made extensive edits throughout the manuscript to address reviewer concerns and comments. Moreover we have added a range of additional analyses to the supplemental files to clarify multiple aspects of the analysis, and expanded several sections of the introduction and discussion.

*** General changes

i. We have greatly expanded discussion of the entomological and epidemiological setting to contextualize this intervention in an area of residual malaria transmission with very complex anopheline populations.
ii.. We have redirected much of the discussion to highlight implications of this pilot study to inform future related trials.
iii. We became aware of severe limitations in using the Shannon index for non-random samples (as from cattle baited traps), and have consequently reanalyzed the secondary outcomes using the Brillouin index, as per prior work from the medical entomology and ecological literature. However, conclusions from this secondary analysis are unchanged.

****** Addressing reviewers' comments *****

I. Methods
Comment: Reviewer #1: The title of the study is "Measuring effects of ivermectin-treated cattle on malaria vectors in Vietnam: a village randomized trial". My concern is are these mosquitoes really the vectors of human malaria? or do they just happen to bite. All the collections were carried out using cattle bait trap- thus these are mosquitoes that are zoonotic and thus would be responsible for transmission of malaria. I feel at least the title should be changed. There should have been mention of the number of cases in the villages
*** Author Response. We fully appreciate the reviewer's point that all anopheline captures may not be vectors. To address this comment, we have added the word "potential" to the title of our manuscript, and have expanded discussion of the entomological context. We have also expanded the introduction and added an epidemiological overview, including reported malaria cases per village.

Comment: Reviewer #2: Unsure of the true power of the study. Though it should not impede publication, a more honest discussion in the abstract world help readers understand the frailties.
*** Author Response. To clarify the power in the abstract, we have added the sentence, "This intervention was powered to detect a 50% reduction in total nightly Anopheles spp. vector catches from cattle-baited traps." To the methods section within the abstract, and have added an separate section on study power.

Comment: Reviewer #3: -Are the objectives of the study clearly articulated with a clear testable hypothesis stated?
Yes, but additional analyses are warranted. Should perform assessment by Anopheles species with enough subjects for analysis.

*** Author Response. To demonstrate the impact of IVM on specific species, we have included an additional figure showing changes in specific mosquito species over the study time period. For this figure, the six most prevalent Anopheles species were included. All species in this figure have at least 100 captures over the twelve individual trapping nights. As shown in the figure, there does not appear to be a dramatic change in trend for any species. We have additionally run a two-sample t-test to compare the difference in mosquito captures during the pre vs. post period across treatment vs. control villages. For each of the six most prevalent species, there was not a statistically significant difference in mean number of catches in the treated vs. control villages. The results of these statistical test are shown in Supplemental Table 3.

-Is the sample size sufficient to ensure adequate power to address the hypothesis being tested?

Yes, according to their assumptions. It is not clear where the 50% reduction in Anopheles catches comes from, explain. There should be a section for "Sample Size" separated from the "Study Design"
*** Author Response. We have added a separate "sample size" section, and delineate a range of entomological studies that use a 50% reduction for design.

The Methods require more clarification. In particular explanation of the number of collection nights before and after the intervention that the mosquito collections occurred, figures suggest that collections occurred every other night. Was only one cow/location used on each collection night? Also, these are collection nights, do not refer to them as days. What does "swept with hand aspirators" mean, clarify. Clarify what "processing" occurred with the saved mosquitoes? Is this not a cluster randomized trial with the unit of randomization as the village? If yes, then change language, if not, then explain why the "village-based randomized controlled trial" is not a CRT. State the Exclusion Criteria as such, what about lactating cattle?

*** Author Response. We thank the reviewer for these important comments. To address these, the following additions and edits have been made:
1. Using the three most prevalent mosquito species, species-specific analyses were conducted. These are presented in the supplement.
2. We have explained the 50% reduction assumption in a section on power calculation in the methods
3. We have added a new section entitled sample size. This section includes information on the number of species expected to be captured, as well as information about the types of vectors present in Vietnam.
4. We have added further details on the specifics of nightly trapping.
5. We have changed "days" to "trapping nights" throughout

6. Re: cluster- versus village-randomzied trial. There is considerable confusion in the literature on this point; we have edited the title to reflect the unti of analysis.

II. Results
Comment: Reviewer #1: The results presented are okay. But would have been useful to have at least a supplementary table showing the numbers for the different species in the various villages before and after.
*** Author Response. We appreciate this point, and have added in a table (supplemental info) showing the raw counts of mosquitoes by species captured during the pre- and post periods using cattle baited traps within each village.

Comment: Reviewer #3:
-Are the results clearly and completely presented?
What were the number of cattle for each village, include the control villages as well. Is there human census data? Were there buffalo present in the villages, if so report. What are "units of ivermectin", is this ml, if so explain.
*** Author Response. We have added this information to table 1, along with API data from 2018 in the text and in the SI. We have also clarified in the methods that cattle were censused in treatment villages only, after randomization.

-Are the figures (Tables, Images) of sufficient quality for clarity?
Dates should not be used in Figures 2-4, instead label each collection night as time before or after the intervention. The numbers in Figure 5 circles do not match the actual collection nights post intervention. Figure 5 is not a common presentation for vector control trials, an explanation of what would be expected impacts of a successful vector intervention on Evenness and Richness. An additional figure should be provided which is an aerial view of the village distribution with scale bars for distance, this will help the reader understand potential spillover concerns better.
*** Author Response.
1. We have included a supplemental table including the number of cattle for each village.
2. There were no buffalo present; this has been clarified.
3. We have removed calendar dates from all figures.
4. We have included an explanation of some potential expected results on indexes from a successful intervention.
6. We have added a map view of villages with scale bar to the supplemental info, and expanded the discussion on spacing between villages to address these concerns.

III. Conclusions
Reviewer #1: The authors have mentioned that better results would be obtained if 80% of the cows were treated. However, I feel that malaria cases should have been included to show the effects since most of the Anophelines are not the major vectors.
*** Author Response. In this area of residual malaria transmission, all potential vectors are important for elimination. We have expanded discussion of this aspect of the study, and added reference to 2021 manuscript (same co-investigators and entomological teams) addressing

these issues, and made explicit where the 80% target came from. Case burdens are too low to allow for any epidemiological outcomes.

Reviewer #3: -Are the conclusions supported by the data presented?
The duration of IVM activity would have no impact on your analyses. Published results to date suggest one to three effects on mosquito mortality, so how would your 10 day window post intervention be affected by this. You should summarize results of previous cattle treatment studies in the Introduction. LongRange is eprinomectin, not ivermectin, at least cite a long-lasting ivermectin formulation.
*** Author Response. We have addressed these issues in the text.

Crossover section would be enhanced if a map of the site was provided. The argument that mosquitoes that ingested ivermectin "need to rest to digest the bloodmeal" is not correct. Sub-lethal ivermectin concentrations reduces locomotor function (Sampaio et al. 2017), and delays time to refeed (Kobylinski et al. 2010, 2017), this argument should be updated. The argument of anthropophilic mosquitoes impacting the results begs a breakdown of analysis by Anopheles species. Also discuss the species predilection for feeding on humans or cattle. It is not clear how you would know or not know if mosquitoes were selecting alternative hosts if you were not performing human landing collections.
*** Author Response. We have added information about HLC and CDC traps, and updated discussion of feeding preferences throughout the text.

Previously in paper it was stated cattle kept near the house, so it is not clear how cattle movement would impact in this situation.
*** Author Response. We have added a section of cattle rearing practices in these villages to contextualize the intervention, including daily cattle movements.

My understanding from your methods is that there is one cow used per trap night per village. If there are 120-629 cattle in the treated village then I see no way that "extensive" trapping of mosquitoes could have occurred. The SolarMal citation specifically placed a density of traps to intentionally reduce mosquito populations. This entire concept of "extensive" mosquito trapping seems highly unlikely and should be removed.
*** Author Response. we appreciate the reviewer’s consideration of this issue, and the reference to the SolarMal study has been removed.

-Are the limitations of analysis clearly described?
Not really. Major limitations would be a lack of baseline data to understand how abiotic factors such as moon phase impact the mosquito populations. Repeated ivermectin administrations across the duration of the malaria season could help to minimize this issue.
It is not clear what they mean by "dose-density" it seems they are referring to varying amounts of coverage and not escalating dose levels of ivermectin, this should be clarified.
*** Author Response. we have clarified discussion of lunar phases; multiple treatments; and explicated our (somewhat non-standard) use of “dose-density” relative to total anopheline density.

IV. Editorial and Data Presentation Modifications
Comment: Reviewer #1: I find it very difficult to make a decision. I agree a lot of work has gone into the study and thus at least make an attempt to change the title of the manuscript.
*** Author Response. We greatly appreciate the acknowledgement of the work inherent in this study. We have edited the title to reflect concerns about potential vs definitive malaria vectors and have added an explicit section to clarify the complex entomological context.

Comment: Reviewer #3: Introduction mentions "peri-domestic vector feeding is common" but most GMS malaria transmission occurs in the forest where the efficient vectors tend to reside, so these points seem rather at odds for malaria control, please explain.
*** Author Response. This is an important point and the importance of peridomestic vector control in elimination settings has been made explicit; and the ento-epidemiological context including forest-fringe malaria has been explicitly discussed.

Ivermectin lipophilicity and metabolisation are two separate concepts. Metabolites have been identified from humans and shown to have mosquito-lethal effect (Kobylinski et al. 2023, Kern et al. 2023) so not sure the point of mentioning these issues? And they should have proper citations.
*** Author Response. We appreciate the reviewer highlighting this incorrect linkage; and have corrected it. However, we have not included further discussion of the many bioactive metabolites as it is beyond the scope of this work.

Ivermectin half-life when injected into cattle is >2 days so not sure how this is defined as short. Citation #12 does not reference mosquitoes. The cattle/Anopheles experiments should be described in better detail, so the reader understands duration of efficacy with a 200 ug/kg dose. Why would administration of ivermectin to cattle be ideal for targeting GMS mosquito anthropophilic behavior?
*** Author Response. We have clarified the use of standard veterinary health dosages; and the importance of peridomestic vector control in these elimination settings has been made explicit in both the introduction and in the discussion.

V. Summary and General Comments
Reviewer #1: Perhaps human landing collection should also have been carried out during this study to indicate if the numbers of mosquitoes coming to bite humans were reduced. This will also provide some comparison as to the different species of mosquitoes biting human/cattle
*** Author Response. Concurrent human landing catches and CDC traps were included in this study for all trapping-nights. This has been clarified, and these data added to text and new tables added in the SI.

Reviewer #2: In this paper, the results of the first field trial on the impacts of ivermectin treatment in cattle on mosquitoes are presented. The authors present results on the total number of mosquitoes caught per night across six sampling nights, both before and after the intervention across six villages (3 treatment, 3 control) and also measure changes in species

diversity across the study period. The authors do not report a significant difference between the total number of mosquitoes caught between the control and intervention arm. There is a significant difference in the species diversity index between certain villages, but this is not consistent across the intervention/control arms.
*** Author Response. NA.

Analysis on number of mosquitoes caught between arms
It is possible that the study design may have impacted the ability to detect a significant difference between the control and intervention arm. The authors describe the methodology used to estimate the power of the study, assuming approximately 35 catches per night from a cattle-baited trap (derived from CBT data in Cambodia) and a 50% reduction in mosquito nightly catches in the intervention arm. This seems very optimistic. The authors have not justified the case for borrowing information on CBTs from the Cambodian setting, so it would be helpful to confirm that similar Anopheles species compositions and feeding behaviours are found in Cambodia.
*** Author Response. These are important issues, and a new section on the entomological context has been added.

Furthermore, the data presented in Figure 2 shows that the nightly catches in Vietnam often exceeded 35. No justification is provided for assuming that the between village variance was 0.1, the SD of trapping rates being 20 or the distribution used. Given this, and the heterogeneity in monthly catches per night both within and between villages, the study is likely underpowered. This observed low power is often seen in mosquito experiments where conditions can change substantially and should not preclude publication as there is much within the manuscript that is interesting. This is through no fault of the authors, as a lack of baseline data is often a problem, though I do think they were overly optimistic in the assumptions that went into their power calculations.
*** Author Response. We have expanded the study design power to explore these issues in greater detail, and directly address these limitations within the discussion.

Nevertheless, it is essential that the authors are up front about the actual power the study achieved, so that readers who might not be aware of the problem do not overly interpret results. In such circumstances it is standard practice to calculate the observed power and report it along with the results. This may strengthen the Discussion section of the paper, in terms of ruling-in/out the possibility of the study being underpowered.
*** Author Response. We greatly appreciate the reviewer's suggestions and consideration of these issues. To address this gap in presentation of results, we have added a detailed section in the supplemental information where we delineate power for a range of different coefficient of variation scenarios consistent with our field data to inform future related studies.

Respectfully, the calculation of post-hoc/observed power cannot add any additional information to this study, or to any study with a non-significant outcome, as the two measures are directly linked. From a highly cited paper in the American Statistician (2001), "Observed power can never fulfill the goals of its advocates because the observed significance level of a test ("p

value") also determines the observed power; for any test the observed power is a 1:1 function of the p value." https://www.jstor.org/stable/2685525

If the study is underpowered the authors should consider including this within the abstract so that those that just read this know not to interpret the lack of difference between arms as a failure of the intervention. Certainly, I would consider removing the statement "it was well-powered for primary outcomes" from the discussion, which as I see it has no basis and will confuse interpretation.
*** Author Response. We appreciate this critically important point, and have removed this phrase, as well as expanded sections in both the abstract and discussion addressing limitations of the study's ability to quantify impacts of ivermectin in this setting.

It would also be helpful if the authors provided the rationale behind their sampling strategy and how it is linked to their understanding of the biological impact of ivermectin treatment in cattle on mosquitoes. The ability to collect mosquitoes by the CBT is influenced by the cattle feeding rate of the mosquitoes and the mosquito life cycle; if mosquitoes are feeding approximately once every three days and certain proportion of these feeds are on cattle, the number of mosquitoes that will be caught in the trap will likely be highly variable.
*** Author Response. We have expanded discussion of the CBT design and expected outcomes throughout the text to address these issues.

The authors used generalised estimating equations (GEEs) to compare the number of mosquitoes caught between the intervention and control arms. However, GEEs are only reliable when there are enough clusters in each arm. Given the heterogeneity in catches within each village (and across arms), the use of GEEs to detect a difference between the intervention and control arm may be limited. Whilst the authors have included error-clustering at the village-level in the model, the small number of villages in the trial may affect the fit of the model to the data. It would be useful to see a figure of the fit of the GEE to the data.
*** Author Response. A plot of model residuals has been added to the SI, along with a range of alternative model specifications and a differences-in-differences plot to more fully explore the modeling assumptions.

The GEE also has a Poisson distribution, however given that the trapping data from Kong Pa using CDC light traps "found a mean of 3.5 vectors/night (median 1; SD = 6.5)" the data is very over-dispersed and the Poisson distribution is unlikely going to be able to capture this adequately given the very high number of mosquitoes caught per night shown in Figure 2. The authors should further justify their use of the Poisson distribution over, for example, a negative binomial distribution which would capture this over-dispersion even when the number of nightly catches is high.
*** Author Response. The pre-specified model for analysis was neg binomial; this has been made explicit, and the distribution made clear throughout.

Species diversity analysis
The analysis on the number of mosquitoes caught in nightly traps is presented at the arm-level (i.e. the counts are compared between the intervention and control arm), the authors should justify why the analysis in the main-text focuses on the within-cluster differences between the Shannon diversity index. The paper would benefit from having results presented at the same unit, either between arms or between villages, or both. Indeed, looking at the difference between villages the changes seem somewhat stochastic, so could not a clearer picture might be observed by doing the analysis at the arm level.
*** Author Response. This is a very important point to ensure clarity in the results; all plots have been updated to have harmonized reporting units and unified legends throughout the revised manuscript.

The authors identified a significant difference between the Shannon Diversity Index in the pre-intervention arm vs post-intervention arm in some villages. However, the p value was only below 0.05 in two of the treatment villages. Furthermore, in one of the treatment villages (H Lang), the increase in the relative abundance of An. vagus is apparent from day 6 of the pre-intervention collections, suggesting that the detected shift in species composition may not be due to the intervention at all.
*** Author Response. As noted in the overview to revisions, we have refined both these analysis and discussion related to the diversity indexes to address this point, and other issues with the secondary analysis of anopheline population composition.

In the Discussion, the authors comment on the possibility of spillover of vectors between the intervention and control arms and that this may impact the ability to detect a significant difference in the number of mosquitoes caught per arm, however little evidence is provided to comment on the extent to which this is influencing the results. The species composition shown in Figure 3 shows that the sites are quite different in terms of mosquito species. It would be helpful if the authors included a map (in the supplementary material) of the study villages as this would help to ascertain the likelihood of vector spillover between sites. For example, if Oi Dak and Oi Jit (have similar species compositions according to Figure 3) are sufficiently close together, then it is likely that vector spillover is occurring (given mosquito flight range).
*** Author Response. We appreciate this important gap; a map of study areas has been added to the SI, and fuller consideration of spillover effects (and additional references on this topic) have been added to address these issues related to vector diversity and potential spillover.

Other points for Discussion section
The epidemiological impact of ivermectin on malaria transmission can vary between study sites due to mosquito feeding behaviour and ecology, susceptibility of the mosquito species to ivermectin and the combination of vector control tools already in place. Whilst the authors have commented on the species composition and feeding behaviour of the dominant vector species in this study site, little/no information is provided on the susceptibility of the species to ivermectin (Chaccour et al. (2023) comments on the differences in susceptibility to ivermectin between Anopheles species). It would be helpful if the authors mentioned in the Discussion whether or not this data is available for the species found in Viet Nam, as they have commented

on the potentially short half-life of ivermectin and how this may have influenced the results of the trial.
*** Author Response. We appreciate this point, and have added the (limited) data on differential susceptibility in local anophelines to the text; and added this important reference.

Furthermore, it is not clear whether other control tools are in place e.g. are cattle owners treating cattle with insecticide or spraying cattle shelters with a repellent (as is done in some locations). Readers also need to be made aware that the trial would need a different endpoint to confirm whether or not the treatment of cattle with ivermectin will have an impact on malaria epidemiology, as this is not currently stated.
*** Author Response. We have expanded discussion on current vector control and limitations of epidemiological endpoints in this very low-transmission setting.

Minor points.
Figure 4 should state what the solid and dashed lines are. Though it is consistent with other figures, it should be stand alone.
*** Author Response. We have edited all figures to be consistent, and harmonized legends throughout this revised manuscript.

Reference section requires work to make consistent. For example, some journals are abbreviated and others not.
*** Author Response. We appreciate this point, and all citations have been adjusted to be consistent with PLOS style guidelines.

Some statements and abstract and the discussion require further justification. For example, why should "Future studies should devise plans to treat at least 80% of the cattle in the village in order to best evaluate results". Clearly this was not possible within this study, so might not be possible when interventions are implemented at scale, so why the requisite for >80%.
*** Author Response. We have added references for precedent for this 80% target to address this lapse.

References
Chaccour, C., Casellas, A., Hammann, F., Ruiz-Castillo, P., Nicolas, P., Montaña, J., Mael, M., Selvaraj, P., Duthaler, U., Mrema, S., Kakolwa, M., Lyimo, I., Okumu, F., Marathe, A., Schürch, R., Elobolobo, E., Sacoor, C., Saute, F., Xia, K., ... Rabinovich, N. R. (2023). BOHEMIA: Broad One Health Endectocide-based Malaria Intervention in Africa—a phase III cluster-randomized, open-label, clinical trial to study the safety and efficacy of ivermectin mass drug administration to reduce malaria transmission in two African settings. Trials, 24(1), 1–16. https://doi.org/10.1186/S13063-023-07098-2 /PEER-REVIEW

Comment: Reviewer #3: Statements on data availability should be made
*** Author Response. A link to the study data repository has been added, https://osf.io/eh7sc/
